# An exploration of unusual antimicrobial resistance phenotypes in Salmonella Typhi from Blantyre, Malawi reveals the ongoing role of IncHI1 plasmids

**DOI:** 10.12688/gatesopenres.16311.1

**Published:** 2024-12-23

**Authors:** Allan Zuza, Alexander M. Wailan, Catherine Anscombe, Nicholas A. Feasey, Eva Heinz

**Affiliations:** 1 Malawi Liverpool Clinical Research Program, Kamuzu University of Health Sciences, Blantyre, Malawi; 2 Wellcome Sanger Institute, Hinxton, England, UK; 3 University of St Andrews, St Andrews, Scotland, UK; 4 Liverpool School of Tropical Medicine, Liverpool, England, UK; 5 University of Strathclyde, Glasgow, Scotland, UK; 6 Liverpool School of Tropical Medicine, Liverpool, England, UK

**Keywords:** Antimicrobial resistance, Typhoid, H58, drug resistance, gene loss

## Abstract

Typhoid fever is a significant public health problem endemic in Southeast Asia and Sub-Saharan Africa. Antimicrobial treatment of typhoid is however threatened by the increasing prevalence of antimicrobial resistant (AMR) *S.* Typhi, especially in the globally successful lineage (4.3.1) which has rapidly spread in East and Southern Africa. AMR elements can be found either on plasmids or in one of the three chromosomal integration sites, and there is variability of this across the lineage. Several previous studies with Malawian isolates indicated a clonal, locally spreading lineage with chromosomally integrated resistance genes. In a recent study however we noted three isolates with predicted resistance genes unusual for the region, and we here present the resolved genomes of these isolates using long- and short-read sequencing. Our work shows that these isolates are potentially imported cases, most closely related to the recently described sub-lineage 4.3.1.EA1, although they encode IncHI1 plasmids with reduced resistance gene repertoire compared to the main IncHI1 plasmids spreading in East Africa. Similar reduced plasmids were reported in a recent large-scale study in five isolates from Tanzania, highlighting the urgency for better coverage of the African continent in genome studies to better understand the dynamics of these potentially co-circulating plasmids.

## Introduction

*Salmonella enterica* subspecies *enterica* serovar Typhi ( *S.* Typhi) is estimated to cause over 11 million typhoid fever cases annually. ^1^ Typhoid caused by *S.* Typhi is endemic in Southeast Asia and Sub-Saharan Africa and a major cause of under-five mortality ^1^ and treatment of infections by *S.* Typhi relies on antimicrobial therapy. The prevalence of *S.* Typhi resistant to first-line antibiotics is rising in Africa and a major cause for concern, although in Asia, there has been re-emerging susceptibility to these agents, further emphasizing the relevance of antimicrobial resistance (AMR) surveillance. ^2^^–^^4^

The spread of AMR genes in *S.* Typhi has been tracked in unprecedented resolution using large-scale whole-genome sequencing surveillance and can clearly be traced to specific lineages that acquired resistance elements and then spread clonally across large areas. Across Africa, there are two main lineages ^5^; in Eastern Africa the spread of resistant *S.* Typhi is mainly driven by the highly successful lineage 4.3.1 (formerly haplotype H58 ^6^^,^^7^) which is originally associated with plasmid IncHI1 and which rapidly spread across the African continent from appr. 2005, ^8^ whilst in Western Africa, the genotypes 2.3.2 and 3.1.1 dominate. ^5^ Recent studies place the origin of 4.3.1 to South East Asia, ^9^ and highlight it may have potential advantage over other lineages not only by its AMR gene repertoire but also through better intracellular survival, which was observed in phenotypic assays. ^10^

The resistance genes commonly found in *S.* Typhi confer resistance to aminopenicillins ( *bla*_TEM-1_), chloramphenicol ( *catA1*), both constituents of co-trimoxazole ( *dfrA, sul1*/ *sul2*), and streptomycin ( *strAB*) as well as tetracycline ( *tetA*) and can be located either on plasmids or on one of three chromosomal sites through the integration of a Tn *2670*-like element, ^5^^,^^8^^,^^11^^,^^12^ and there is variability of this within and between different lineages. It is likely that the acquisition of the IncHI1 plasmid happened only once, and this event was one of the key factor in the success of 4.3.1 globally. ^9^ Previous studies with Malawian isolates have shown the presence of AMR genes in the 4.3.1 haplotype through chromosomal integration ^11^^,^^13^ and a clonal, locally spreading lineage. The IncHI1 plasmid is known to have substantial variability and is hypothesized to have acquired several resistance gene cassettes through step-wise insertions in an insertion hot-spot. ^14^^,^^15^ Most 4.3.1 isolates in recent large-scale sequencing studies encoding for an IncHI1 plasmid include the core set of resistance genes as described above, which would support the hypothesis that antimicrobial pressure selects for the most resistant lineages which outcompete ones encoding for IncHI1 plasmids encoding fewer resistances. ^5^^,^^14^

In a recent study we noted three isolates with unusual resistance gene profiles indicating partial loss of the main resistance elements; these three were however also predicted to carry IncHI1 plasmids which is unusual in the Malawi context. ^13^ We aimed to resolve the genetic structure of these IncHI1 plasmids and understand the location of the AMR genes as well as the structure of the genome integration site where the dominant lineage found in Malawi carry the resistance elements chromosomally integrated. The unusually low number of resistance genes (for IncHI1-encoding concurrent isolates) might indicate a re-emergence of susceptibility to older drugs which have been reduced in use following the widespread resistance, a phenomenon that has been observed in particular for chloramphenicol in other highly drug-resistant bacteria in sSA. ^16^

To understand the structure and dynamics of this reduced resistance gene repertoire, we performed long-read sequencing to fully resolve the chromosomal and plasmid structure. Our detailed analyses furthermore facilitated establishing whether these were likely imported cases or whether they are part of a lineage spreading in Blantyre carrying the IncHI1 plasmid. We selected four representative isolates, including two with reduced resistance gene profiles and two as comparisons for putative insertion site changes, and report a detailed description of their genome and plasmid structure, as well as comparisons of the plasmids with reference sequences for the *S.* Typhi IncHI1 plasmid commonly found in 4.3.1. These provide valuable insights and a reference for future studies where similar profiles might be observed in the region, and it will be highly relevant to monitor potential increase in re-emergence of sensitivity to first-line drugs that have in the meantime gone out of routine use.

## Methods

### Long-fragment DNA extraction

These bacteria were isolated from the blood of febrile patients attending Queen Elizabeth Central Hospital in Blantyre, Malawi, as part of the quality assured routine diagnostic microbiology service supported by the Malawi Liverpool Wellcome Programme as described previously. ^11^ ERS327391 and ERS207185 were previously sequenced by Feasey *et al* and ERS1509723 and ERS1509734 were sequenced by Gauld *et al.* ^11^^,^^13^ using short-read sequencing only, with ERS1509723 and ERS1509734 predicted resistance gene prediction and IncHI1 replicon, unusual for this region. Isolates were recovered from the MLW sample archive and then plated on nutrient agar (Thermo Scientific Oxoid, United Kingdom) and incubated at 37 °C for 18 hours. A single colony was then transferred into 5 ml of nutrient broth (Thermo Scientific Oxoid, United Kingdom) and incubated for 18 hours at 37 °C. The bacteria cells were then concentrated by centrifugation at 3500 rpm for 30 minutes. Total nucleic acids were extracted using the MasterPure complete DNA and RNA Purification Kit (Bioresearch Technologies, United Kingdom) according to the manufacturer’s instructions.

### Sequencing

The double-stranded DNA was quantified using the Qubit 4.0 (ThermoFisher Scientific Inc.) fluorometer and normalized to 400ng in 7.5 ul using UltraPure™ Distilled Water (Invitrogen, Life Technologies Limited). The normalised DNA was used for library preparation using the rapid barcoding kit (SBQ-RBK004, Oxford Nanopore Technologies plc) following the manufacturer’s instructions. The prepared library was sequenced on an Oxford Nanopore r9.4.1 flow cell on the MinION Mk1C sequencer (Oxford Nanopore Technologies plc). Data acquisition was done by the MinKNOW ^TM^ software (version 20.10.6, Oxford Nanopore Technologies plc). Base calling and demultiplexing using Guppy Basecalling Software, Oxford Nanopore Technologies plc. (version 6.0.7+c7819bc). We used Guppy with the default settings and the “dna_r9.4.1_450bps_sup.cfg” configuration file for the guppy_barcoder. We used the command line argument “--trim_barcodes” for the debarcoding step using guppy_barcoder. For guppy_barcoder we passed “SQK-RBK004” to the “--barcode_kits” command line argument.

### *De novo* assembly

We retrieved the short reads from ENA and performed trimming and filtering using fastp (version 0.23.1--9f2e255. ^17^^,^^18^ The reads derived from nanopore sequencing were trimmed and filtered using Filtlong (version 0.2.0--0c4cbe3) with options “--min_length 1000” and “--keep_percent 90” to discard all reads less than 1kb and remove the worst 10% reads. ^19^ We used FastQC (v0.11.9_cv7) and NanoPlot (version 1.38.1--e303519) with the default settings to check the quality of the filtered short and long reads respectively. ^20^^,^^21^

We performed long-read-first *de novo* assembly using the Trycycler (v0.5.4) assembly pipeline. ^22^ Briefly, we created 16 read subsamples using Trycycler subsample. We created draft assemblies on each of the simulated reads using either Flye (version 2.9.1-b1780), Minipolish (version 0.1.2) and Raven (version 1.5.0) assemblers. ^23^^–^^25^ We used the “--nano-hq” command line argument when generating the Flye assemblies. We run the rest of the assemblers using default settings. We used Any2fasta (version 0.4.2) to convert the gfa formatted output from Minipolish to a fasta file format. ^26^ We used the generated assemblies to create groups of per-replicon clusters using trycycler cluster. ^22^ We then performed manual curation steps using trycycler reconcile, trycycler msa, trycycler partition and trycycler consensus following instructions on ( https://github.com/rrwick/Trycycler/wiki/How-to-run-Trycycler ). ^22^

We polished the assemblies using medaka (1.8.1), Polypolish (version 0.5.0) and POLCA (MaSuRCA version 4.1.0). ^27^^–^^29^ The assembly was first polished with the long reads using medaka with “r941_min_sup_g507” passed to the -m command line argument. ^27^ This polished assembly was further polished using short read data using Polypolish and finally using POLCA. Both Polypolish and POLCA were run using default settings. ^28^^,^^29^

The three additional plasmids of early H58 isolates (ERR1764576 (1990, India), ERR1764585 (1992, India), ERR1764588 (1993, India); ^9^ were assembled from Illumina HiSeq 2500 paired end reads using Spades (Ref. 30; version 4.0.0) with the “--plasmid” flag which launches the plasmidSPAdes pipeline that assembles plasmids from whole genome sequencing data. We used the default K-mer values for the assembly.

### Genotyping, plasmid calling and AMR gene detection

Bacterial genotyping and plasmid detection was done using the GenoTyphi bacterial typing framework. ^6^^,^^31^ We implemented GenoTyphi using Mykrobe v0.12.1 with the default settings and the Typhi panel (version 20221208 ^31^^,^^32^). The json output files were parsed into a csv document using the script “parse_typhi_mykrobe.py” provided at the GenoTyphi repository. ^32^

We then used AMRFinderPlus (software version 3.11.26 and database version 2023-11-15.1) to assess the presence of AMR genes and plasmids in the assembled contigs. ^33^ AMRFinder Plus was run by passing the”–nucleotide –protein –gff –organism –plus” flags. We passed “Salmonella” to the –organism flag. AMRFinderPlus outputs a file with AMR genes and their location on the contigs. The AMR genes are presented in Figure 2. We used ariba (version 2.14.6) using the ARG-ANNOT database to detect AMR genes in the raw reads to annotate the phylogenetic trees. ^33^^,^^34^

### Bacteriophage detection

**Figure 1.**
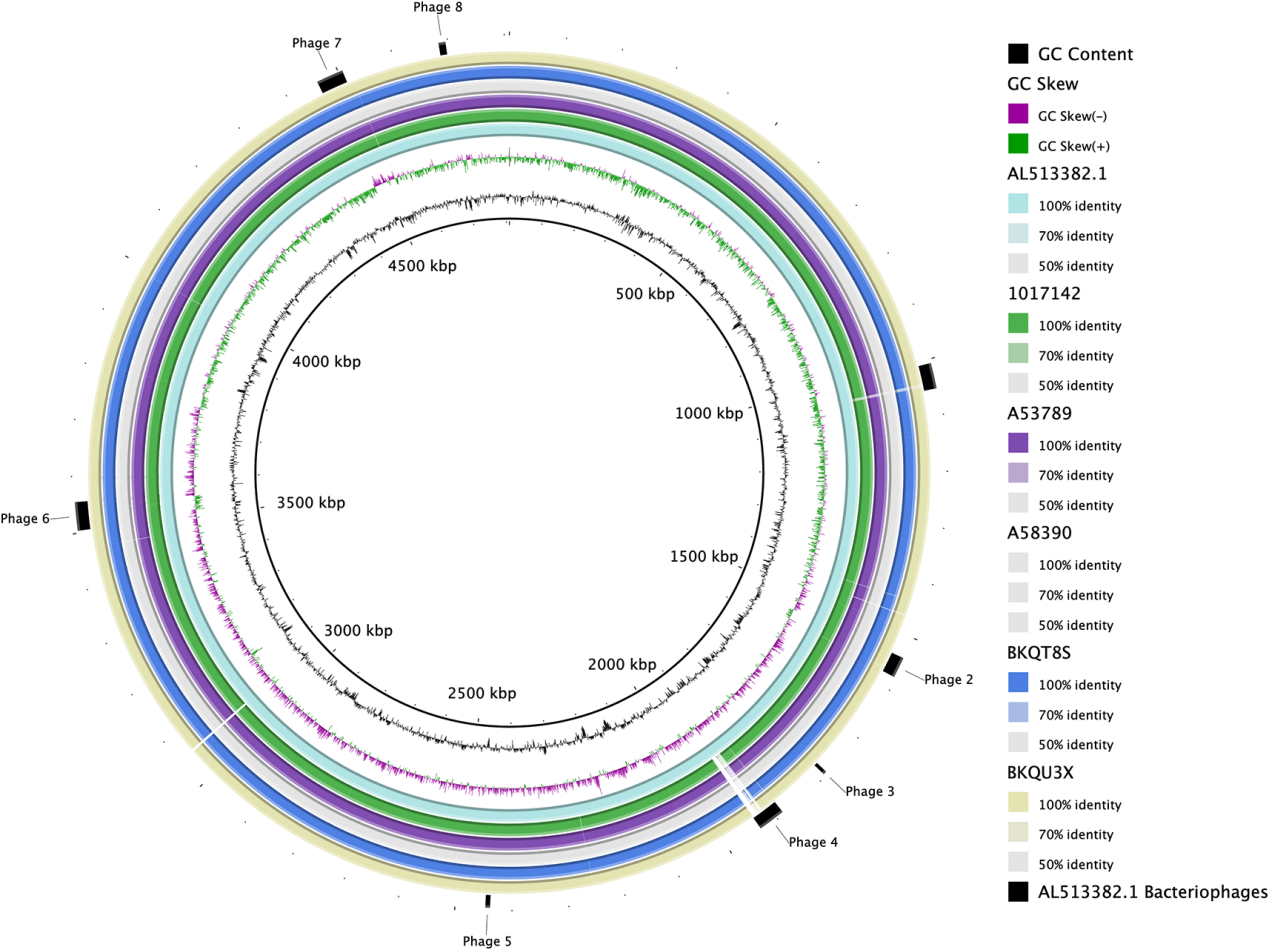
Whole-genome comparison to the CT18 reference. Pairwise BRIG comparison of the hybrid assemblies to the CT18 reference strain as indicated in the legend. The figure also highlights regions containing bacteriophages (outer ring).

**Figure 2.**
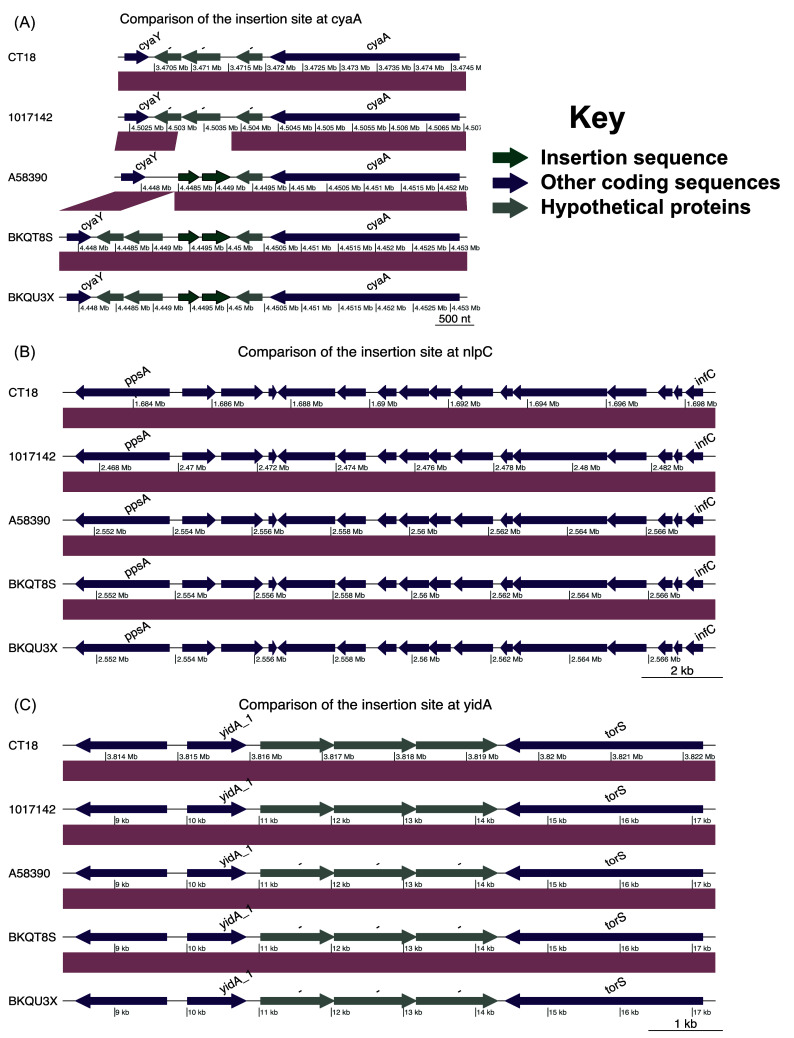
Comparison of the three main described insertion sites for antimicrobial resistance elements. (A) Comparison of the genomic regions showing the introduction of insertion sequences near the *adenylate cyclase* gene at position 3472059 of the CT18 strain. Conservation of the known insertion sites at positions; (B) 3815480 and (C) 1690327 of CT18.

To detect bacteriophages, we used the PHASTER web tool ( https://phaster.ca/). ^35^^,^^36^ We loaded the polished assemblies into the web tool and exported the results as a summary.txt file containing the position of phages in the CT18 chromosome, shown in Figure 1 and Figure 2A.

### Annotation

We performed annotation of the hybrid assemblies using Prokka (version 1.14.6) with the default settings and performed a genus-specific annotation with “Salmonella” passed as an argument to the “-–genus” command line option. ^37^

### Sequence comparison

We used the Artemis Comparison Tool (ACT release 18.2.0) to perform a comparison of assemblies for the isolates sequenced on the MinION platform. ^38^ We visualised the comparison in the Blast Ring Image Generator (BRIG version 0.95 ^39^). Both of these programs used the Basic Local Alignment Tool (BLAST; Nucleotide-Nucleotide BLAST 2.15.0+) to perform nucleotide sequence comparisons. ^40^ For all sequence comparisons, we used the “blastn” option with the default setting for comparing 2 sequences. We used the CT18 reference genome (AL513382.1) as a reference for the BRIG comparison of the chromosome and used the IncHI1 plasmid from the same isolate (AL513383.1) as a reference for the plasmid comparison. ^41^ To assess the presence of AMR genes on the plasmids and parts of the chromosome previously known to contain the insertion of AMR genes, we used the GenoPlotR v 0.8.11 R package which gives a visual comparison of the coding sequences in these regions. ^40^

### Constructing the phylogenetic tree

We constructed a core single-nucleotide polymorphism (SNP) maximum likelihood phylogenetic tree to put the newly assembled isolates in the context of other isolates from Malawi, which uses all conserved sites (i.e. the core genome) when mapping reads against the reference genome. The isolates used in the phylogenetic analysis are from the paper by Gaud *et al.*, where the assembled isolates were first described (7). We used Snippy (version 4.6.0) to perform variant calling and IQ-TREE to construct the phylogenetic tree. We used AL513382.1 as the reference genome for all variant calling steps, snippy-core to create a multiple sequence alignment file with all the variants from the previous step and snp-sites (version 2.5.1) with “-c” argument to extract sites containing exclusively ACGT from the alignment. We also used snp-sites with “-C” to calculate the number of the constant sites which we then used for the “-fconst” argument in IQ-TREE. We performed the IQ-TREE calculation on the alignment with ACGT only generated as described, with the command line parameters “-m GTR+G -bb 1000”.

## Results

### Isolate selection

We performed long-read whole genome sequencing of 4 isolates (ERS327391, ERS207185, ERS1509723, ERS1509734) to better understand unusual antimicrobial resistance gene patterns in 3 isolates from Malawi as observed previously. ^13^ The years of collection for the isolates were between 2010 and 2016. ^13^ Isolates ERS1509723 (BKQT8S) and ERS1509734 (BKQU3X) were selected for their resistance gene pattern, distinct from the rest of the isolates from Malawi from the same period. ^13^ These two isolates are two of only three isolates with IncHI1 plasmids from the same study. ^13^ Isolates ERS327391 (A58390) and ERS207185 (1017142) were selected because they represented isolates at the beginning of the *S.* Typhi outbreak in Blantyre, Malawi. ^11^ Strain A58390 represents one of the first H58 isolates causing illnesses during the *S.* Typhi outbreak. Strain 1017142 represents non-H58 isolates from the same period. ^11^

### Genome analyses

**Figure 3.**
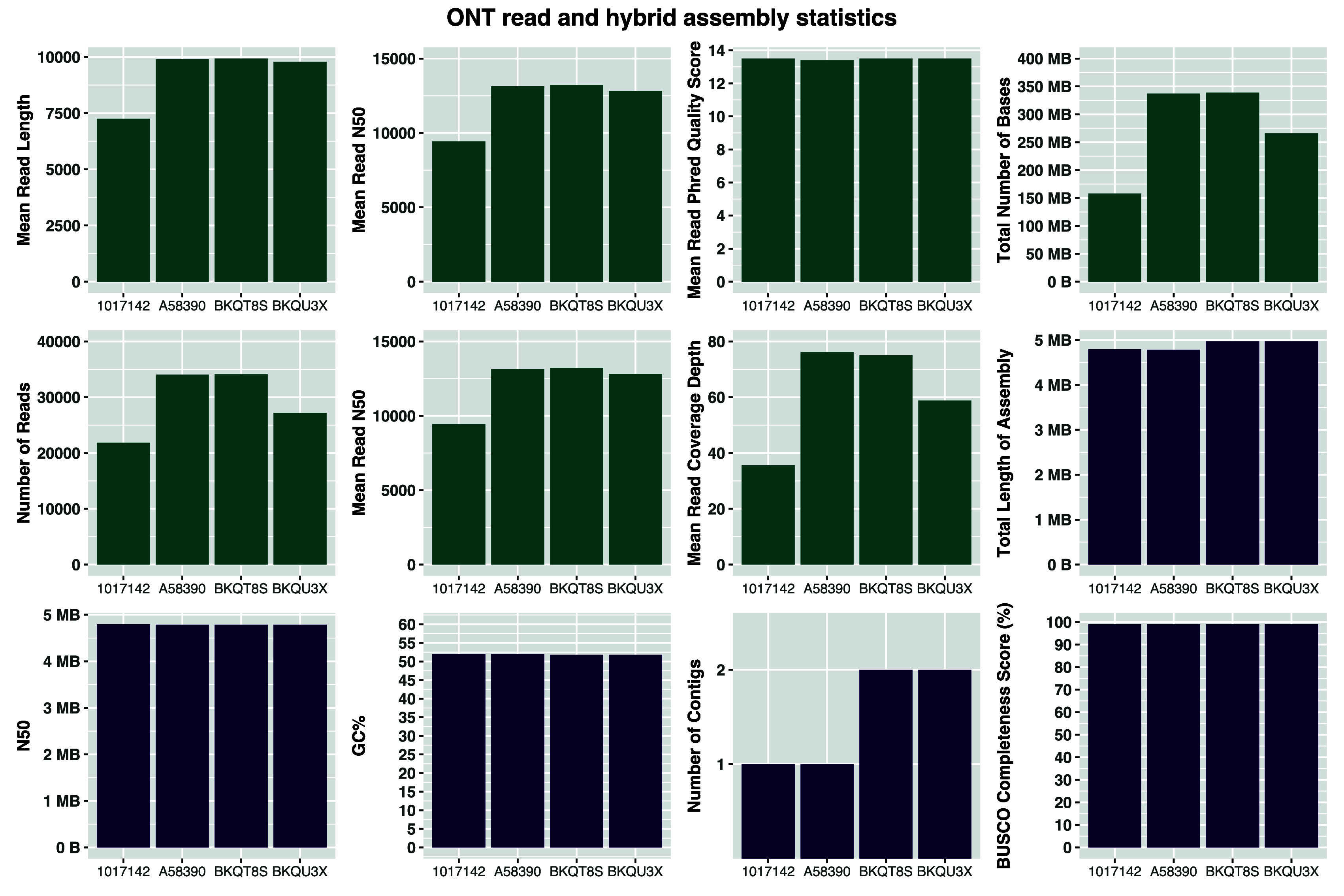
Long-read sequencing quality measures (green bar plots) and hybrid assembly characteristics (violet bar plots).

The nanopore reads for all samples had a mean Phred Quality score of 13.5 with a mean of 29K (21K - 34K) reads per isolate ( Figure 3). We generated a mean 275 Mb reads (158 Mb-338 Mb) with a mean N50 of 12K (9K - 13K) ( Figure 3).

We generated hybrid assemblies of the four isolates, accession numbers and their metadata are presented in Table 1. Two isolates (A58390 and 1017142) assembled to 1 contig each, and the other two (BKQT8S and BKQU3X) assembled to 2 contigs each. All isolates had a 4.5 Mb contig which represented the chromosome of the isolate. The two isolates with 2 contigs, each had a 185 kb contig that was identified as an IncHI1 plasmid.

**Table 1.** Metadata and accessions for the data presented in this study.

Isolate ID	Year of isolation	Source	Short read accession	Long read accession	Assembly accession
1017142	2011	Blood	ERR279139	SRR29530095	CP160102
A58390	2010	-	ERR360832	SRR29530096	CP160059
BKQT8S	2016	Blood	ERR2602821	SRR29530094	CP160060
BKQU3X	2016	Blood	ERR2602832	SRR29530093	CP160062

The assembled genomes had a mean GC% of 52.0 ( Table 2). Three of the four isolates with long reads belong to the lineage 4.3.1 (H58 haplotype); the two isolates with IncHI1 plasmid were assigned sublineage 4.3.1.1EA1 ^42^; the remaining isolate belonging to the lineage 4.1.1 ( Table 2). A pairwise comparison of the assemblies from this study to the *Salmonella* Typhi reference strain CT18 (AL513382.1) shows a high level of sequence conservation as seen in Figure 1.

**Table 2.** Antimicrobial resistance genes and plasmid replicons.

	Oxford nanopore read stats						Assembly stats				
Isolate Id	Number of reads	Mean read length	Mean read quality	Number of bases	Mean length N50	Mean depth	Length of assembly	Gc Percent	Number of contigs	N50	Busco completeness score percent
**1017142**	21817	7247.9	13.5	1.58E+08	9435	35.6995	4795235	52.05	1	4795235	99.5
**A58390**	34062	9897.4	13.4	3.37E+08	13141	76.1677	4782592	52.05	1	4782592	99.5
**BKQT8S**	34113	9930.7	13.5	3.39E+08	13210	75.0613	4969415	51.82	2	4783470	99.3
**BKQU3X**	27176	9789.1	13.5	2.66E+08	12816	58.7983	4969405	51.82	2	4783462	99.3

We assessed the known chromosomal insertion sites for AMR genes ( Table 3). The insertion site at position 3472059 of the CT18 strain was conserved only in isolate 1017142. The other three isolates had insertion of IS1 insertion sequences ( Figure 2A), whilst the insertion sites at 3815480 and 1690327 of the CT18 strain were conserved across the isolates ( Figure 2B and C).

**Table 3.** 

		Plasmid replicons				AMR determinants						
Isolate_id	Lineage	IncFIAHI1		IncHI1A	IncHI1BR27	gyrA_S83F	blaTEM-1D		sul2	dfrA14	tetB	aph(3”)-Ib
**A58390**	4.1.1	No		No	No	Yes	No		No	No	No	No
**1017142**	4.3.1.2	No		No	No	No	No		No	No	No	No
**BKQT8S**	4.3.1.1.EA1	Yes		Yes	Yes	No	Yes		Yes	Yes	Yes	Yes
**BKQU3X**	4.3.1.1.EA1	Yes		Yes	Yes	No	Yes		Yes	Yes	Yes	Yes

### Plasmids, AMR and bacteriophages

**Figure 4.**
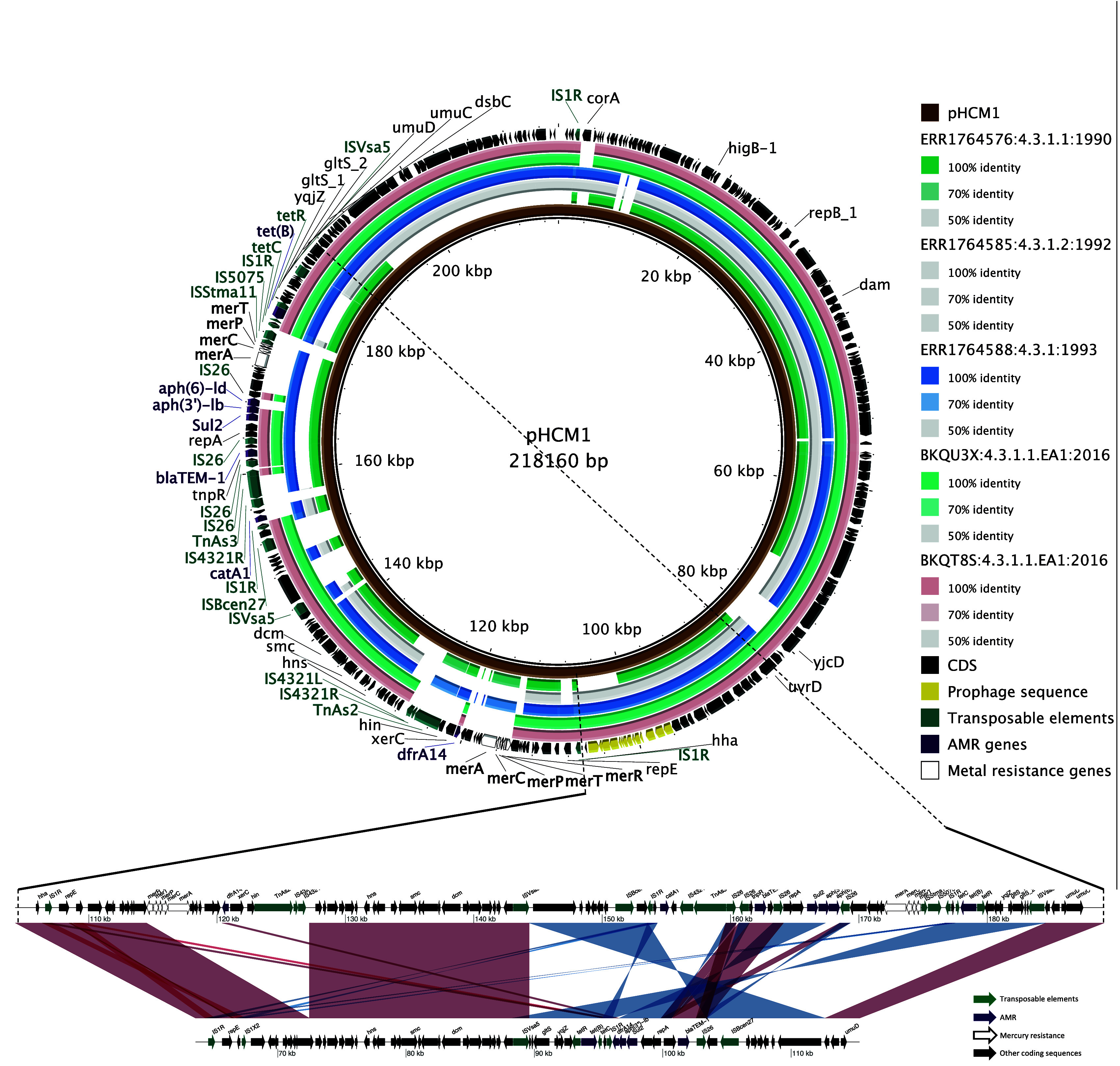
Detailed comparison of the IncHI1 plasmids. A) BLAST comparison of the plasmids from this study to other IncHI1 Plasmids with pHCM1 plasmid from the CT18 reference genome of Salmonella Typhi as a reference. From inside to outside ring: reference sequence pHCM1, ERR1764576 (1990, India), ERR1764585 (1992, India), ERR1764588 (1993, India), plasmid BKQT8S (2016, Malawi), plasmid BKQU3X (2016, Malawi), and a ring of coding sequences in pHCM1 coloured by AMR genes, transposable elements, heavy metal resistance genes, and prophage region on pHCM1. B) BLAST comparison between plasmid of isolate BKQT8S and pHCM1 highlighting the location of IS elements and AMR genes. EA1, East Africa 1; CDS, coding sequences; AMR, antimicrobial resistance.

The plasmids in the isolates BKQT8S and BKQU3X have an average GC content of 45.94% for each isolate. Both isolates had 3 plasmid replicons of the IncHI1 type (IncFIAHI1, IncHI1A and IncHI1BR27; Table 3) which were all on one plasmid. These multi-replicon plasmids are typed as IncHI1 PST2 using pubMLST. ^43^ A blastn comparison using Megablast shows high levels of similarity between the plasmids ( Figure 4A). Comparing the plasmids to the pHCM1 plasmid isolated from the CT18 strain (AL513383.1) and two of the earliest H58 isolates that acquired IncHI1, ERR1764576 (1990, India), ERR1764585 ( 1992, India), ERR1764588 (1993, India), indicates a loss of coding sequences compared to the early isolates and CT18 in an area with antimicrobial resistance determinants ( Figure 4A); small gaps in the early isolates are mainly mobile element sequences, these gaps are potentially derived from the differences in sequencing methods (short-read-only assemblies of the early isolates). The two plasmid sets have a notable difference to pHCM1 in coding sequences in two regions, HCM1.149 - HCM1.175 (region 1) and HCM1.194 - HCM1.252 (region 2). The first region lost 17 coding sequences, five of these are associated with a mercury resistance operon and one AMR gene ( *dhrA14*) has relocated to a different area of the plasmids from our study. The second region has been inverted in the Malawian plasmids. The inverted region lost 13 coding sequences, reducing it in size from ~40kb to ~20kb. The missing genes include mercury resistance genes and importantly the two AMR genes *catA1* and *aph−Id*. ^8^ These two AMR genes are responsible for resistance to chloramphenicol and aminoglycosides respectively. Our plasmid maintained aminoglycosides resistance genotype by maintaining a copy of *aph(3”)−Ib* gene *.* The AMR genes are highlighted in the ring showing the coding sequences of the reference plasmid ( Figure 4A). Figure 4B further highlights the region in comparison to the pHCM1 plasmid.

Neither lineage 4.1.1 isolate 1017142 nor the 4.3.1 isolate A58390 carried any acquired AMR genes, however there was a *gyrA_S83F* point mutation in A58390 which is associated with reduced susceptibility to fluoroquinolones. This was the only isolate with mutations in the DNA gyrase. The other two isolates each carried acquired resistance genes ( *bla*_TEM-1d_, *sul2*, *aph(3”)-lb*, *dfrA14* and *tet(B)*) on the plasmid which are known to cause resistance to beta-lactams, sulfonamides, aminoglycoside, trimethoprim and tetracyclines.

**Figure 5.**
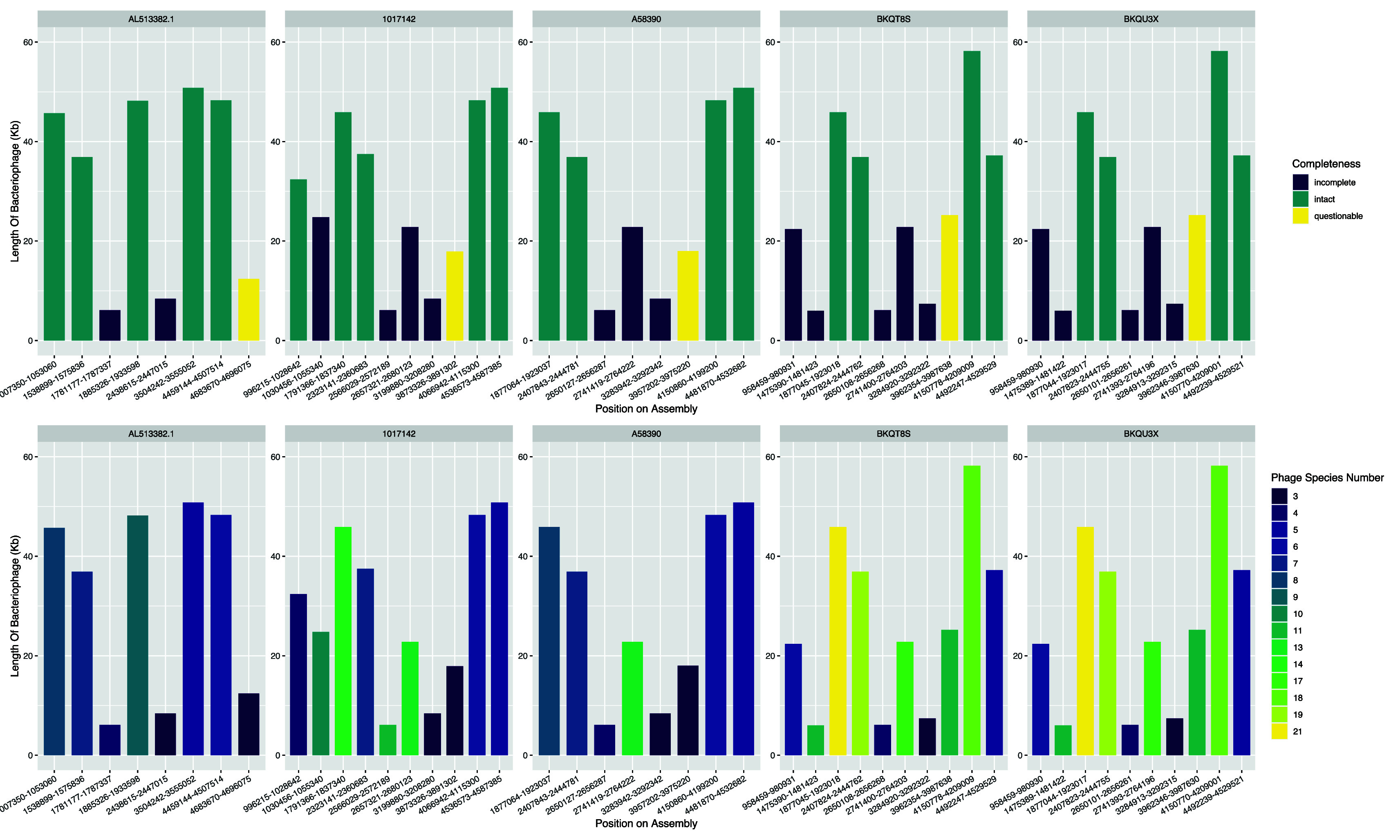
Predicted bacteriophage regions. A) Bacteriophage regions on the assemblies. Each column indicates the length of bacteriophage at the position in the CT18 genome colored by the completeness of the bacteriophage at the region. B) Each column indicates the length of bacteriophage at the position in the CT18 genome colored by the estimated species numbers (bottom row).

Using PHASTER to define phage elements on the assemblies reveals at least four intact bacteriophages, one putative phage call and several incomplete bacteriophages per isolate Figure 5. Isolates BKQU3X and BKQT8S show similarity in the number of bacteriophage species per region and the completeness of the bacteriophages in these regions ( Figure 5). Both isolates contain phages similar in composition and size ( Figure 5B).

### Phylogenetic analysis

**Figure 6.**
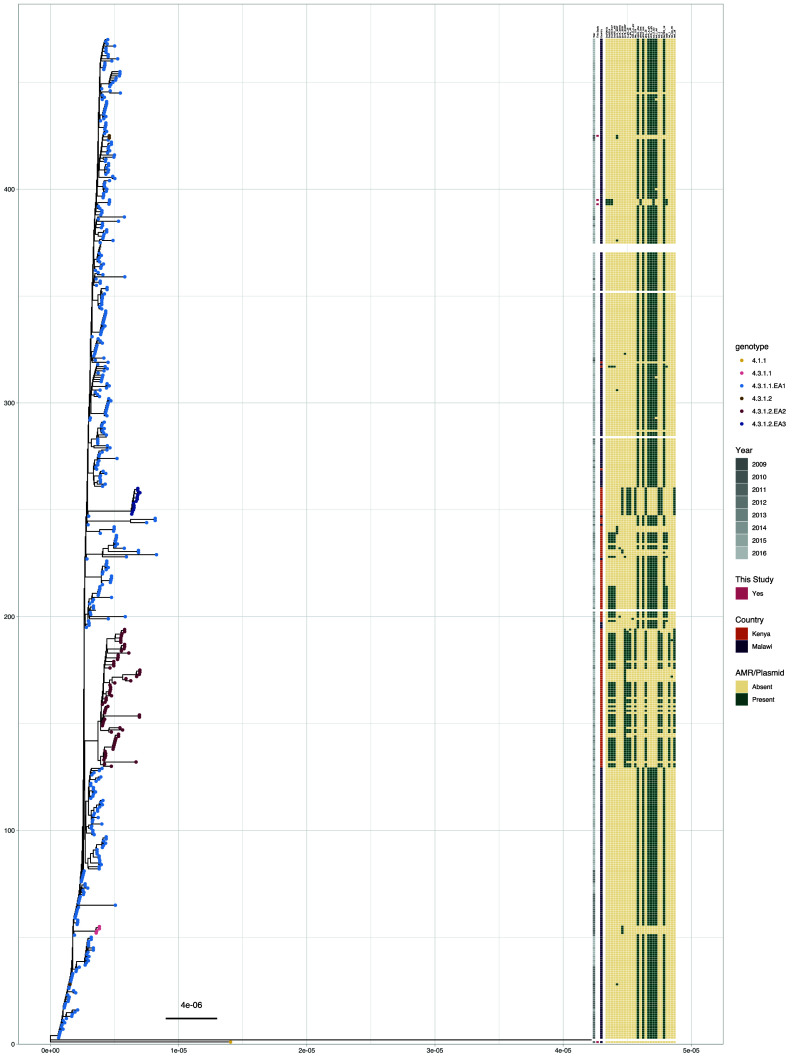
Phylogenetic analysis of the isolates in a wider context. A core-SNP maximum likelihood phylogenetic tree of isolates from Malawi into context by including isolates from Kenya from the same period. The tips are coloured by the genotype of the isolate according to the GenoTyphi typing scheme. The bars indicate the year of isolation, and origin of the samples. The remaining bars show the presence of plasmid replicons in the samples, quinolone point mutations and antimicrobial resistance genes in the isolates as identified by ariba, as indicated by the legends.

Performing a phylogenetic analysis highlights the 4.3.1.1.EA1 plasmid and AMR profiles ( Figure 6). Most of the 4.3.1.1.EA1 isolates do not carry plasmids. Those which carry plasmids seem to be in two plasmid combinations, with isolates from this study carrying the plasmid replicon Inc-FIAHI1 which is missing in the isolates from Kenya, while those from Kenya carry the plasmid replicon Inc-HI1-ST6 which is not present in the isolates from Malawi, indicating this was not a direct import event of the lineage from Kenya via traveller. Isolates with plasmids in the Kenyan collection also have additional AMR genes compared to the rest of the 4.3.1.1.EA1 lineage. Isolates which are not 4.3.1.1.EA1 are clustered in distinct clades and uniform AMR and plasmid profiles. There are distinct differences in acquired AMR genes between isolates of the lineage 4.3.1.1.EA1 and the other H58 lineages. The acquired AMR genes present in the lineage 4.3.1.1.EA1 are absent in the other H58 lineages and vice versa.

## Discussion

We noticed three incidences of predicted IncHI1 plasmids present in Malawi *S.* Typhi isolates amongst a set of 335 isolates, with different resistance gene repertoires. These three isolates carry *tetB* and *dfrA14* in addition to *sul2, strA,* and *strB* genes which are found in all other isolates carrying AMR genes, but lack the *cat1*, *sul1* and *tetD* genes usually seen on IncHI plasmids carried by *S.* Typhi. Initial analyses of the assemblies confirmed a single chromosome assembled for the two control isolates, whereas the two isolates with predicted IncHI replicons assembled as two molecules, the chromosome and one plasmid, as expected.

The two unusual isolates were further investigated given their reduced resistance gene repertoire compared to the main IncHI1 plasmid and we identified that these carry the PST2 IncHI1 plasmids, which is not commonly seen in the 4.3.1 haplotype. ^44^ Two studies previously observed PST2 in non-4.3.1 haplotypes among travelers from West Africa to Europe during the same period as our isolates. ^45^^,^^47^ This IncHI variant, also with a reduced set of resistance genes and the plasmid replicons as encoded in our isolates (including a truncated IncFI replicon) were reported initially for the IncHI plasmid R27 in *S.* Typhi ^48^; an IncFI replicon (although no IncHI replicon) was also noted in several isolates from Tanzania. ^5^

Whilst our samples are not representative of the dominant local *S.* Typhi lineage which has integrated chromosomal resistance, it is interesting to see that this reduced IncHI plasmid type is extant in parts of south-East Africa and embedded in the highly successful 3.4.1.EA1 lineage. This indicates that selection for the larger resistance plasmid might not be as strong as initially assumed ^5^ or might even be disadvantageous in specific settings given its larger size, a trend that has recently been suggested in a large-scale analysis of 13,000 Typhi genomes where increase in multi-drug resistance was correlating to loss of IncHI indicating that chromosomal integration is advantageous over plasmid carriage. ^49^

As the dominant *S.* Typhi population in Malawi encodes the resistance genes at a chromosomal integration site, we investigated these sites in the isolates with IncHI1 plasmids for scars or IS elements that would indicate movement of resistance genes between the plasmid and the chromosome in these isolates. ^12^^,^^50^ We found the insertion of coding sequences and transposases at these chromosomal sites which might suggest the beginning of chromosomal integration. However, no isolates with acquired AMR genes both on plasmids and the chromosome were identified, which would represent a snapshot of this process (genes moving from plasmid to the chromosome) in progress. Our data will be a valuable resource for researchers working on comparative genome analyses of *S.* Typhi, in particular in southern Africa, and contribute to improve our understanding of competitive advantages between lineages with different resistance plasmids. It further highlights the dynamic nature of the resistance genes even in the highly conserved, clonal 4.3.1 lineage, and whilst a small sample size, further emphasizes the importance to keep monitoring re-emergence of susceptibility of first-line drugs that have been out of common use for some time.

## Author contributions

Conceptualization: EH, AMW, NAF; Data curation: EH, AZ; Formal analysis: AZ; Funding acquisition: NAF, EH; Investigation: AZ, AMW, EH; Methodology: AZ, EH; Project administration: EH, NAF; Resources: CA, NAF; Software: AZ, EH; Supervision: NAF, EH; Validation: AZ; Visualization: AZ; Writing-original draft: AZ; Writing-review and editing: AZ, NAF, EH. All authors read and approved the final manuscript.

## Data Availability

Raw sequence data is accessible under project ID PRJNA1127853 at SRA and are outlined in Table 1. Assemblies were submitted to GenBank and are accessible under project ID PRJNA1127853, the individual accessions are presented in Table 1. Sequence Read Archive SRA: Raw sequence data. Accession number PRJNA1127853; (Zuza et al. 2024). https://www.ncbi.nlm.nih.gov/bioproject/PRJNA1127853 Genbank: Assembled genomes. Accession number PRJNA1127853; (Zuza et al. 2024). https://www.ncbi.nlm.nih.gov/bioproject/PRJNA1127853
